# Validation of Administrative Data and Timing of Point Prevalence Surveys for Antibiotic Monitoring

**DOI:** 10.1001/jamanetworkopen.2024.35127

**Published:** 2024-09-24

**Authors:** Riccardo Boracchini, Giulia Brigadoi, Elisa Barbieri, Cecilia Liberati, Sara Rossin, Francesca Tesser, Lorenzo Chiusaroli, Giulia Camilla Demarin, Linda Maestri, Francesca Tirelli, Carlo Giaquinto, Liviana Da Dalt, Silvia Bressan, Anna Cantarutti, Daniele Donà

**Affiliations:** 1 Laboratory of Healthcare Research and Pharmacoepidemiology, Division of Biostatistics, Epidemiology, and Public Health, Department of Statistics and Quantitative Methods, University of Milano-Bicocca, Milan, Italy; 2 Division of Pediatric Infectious Diseases, Department of Women’s and Children’s Health, University of Padua, Padua, Italy; 3 Pediatric Emergency Department, University of Padua, Padua, Italy; 4 Department of Women’s and Children’s Health, University of Padua, Padua, Italy

## Abstract

**Importance:**

Point prevalence surveys (PPSs) are used globally to collect data on antibiotic prescriptions. However, the optimal frequency for data collection to ensure comprehensive understanding of antibiotic use and to target and monitor stewardship interventions remains unknown.

**Objective:**

To identify the optimal frequency for collecting data on antibiotic use among the pediatric population through PPSs leveraging administrative data.

**Design, Setting, and Participants:**

This prognostic study used a cross-sectional validation approach and was conducted in pediatric outpatient and inpatient settings in the Veneto region of Italy. Antibiotics were classified according to the World Health Organization Access, Watch and Reserve criteria. Prescribing rates of access antibiotics were analyzed for pediatric inpatients with records dated between October 1, 2014, and December 31, 2022, and outpatients with records dated between January 1, 2010, and December 31, 2022. The study included children younger than 15 years with an antibiotic prescription who were admitted to the pediatric acute care unit or evaluated by a primary care pediatrician. Data analysis was performed from October 2023 to January 2024.

**Main Outcomes and Measures:**

An algorithm was developed to identify optimal time frames for conducting PPSs. This approach sought to minimize the discrepancy between quarterly and yearly PPS results, aiming to accurately estimate annual antibiotic prescribing rates in both inpatient and outpatient settings (primary outcome). External validity of the optimal PPS time frames derived from outpatient data when applied to the inpatient setting was also investigated. Validation involved assessing the effectiveness of administrative data in identifying strategic PPS periods for capturing inpatient antibiotic use patterns (secondary outcome).

**Results:**

This analysis included 106 309 children: 3124 were inpatients (1773 males [56.8%]) and 103 185 were outpatients (53 651 males [52.0%]). A total of 5099 and 474 867 antibiotic prescriptions from inpatients and outpatients were analyzed, respectively. Outpatients tended to be older than inpatients, with a median age of 3.2 (IQR, 1.3-6.3) years vs 2.6 (IQR, 0.6-6.6) years, respectively, and with a lower burden of clinical comorbidities (≥1 comorbidity: 6618 [6.4%] vs 1141 [36.5%], respectively). The algorithm successfully identified distinct time frames within the calendar year from inpatient and outpatient records optimized for PPS data collection. Rates obtained from the quarterly PPS during these identified periods exhibited greater agreement with annual antibiotic prescribing rates (inpatient: *r* = 0.17, *P* < .001; and outpatient: *r* = 0.42, *P* < .001) than those derived from the yearly PPS (inpatient: *r* = 0.04, *P* = .58; and outpatient: *r* = 0.05, *P* = .34), with a Δ reduction of up to 89.8% (where Δ represents the percentage point change in antibiotic prescribing rates). Furthermore, the optimal PPS time frames gleaned from the outpatient data demonstrated robust applicability to the inpatient setting, yielding comparable results in both scenarios.

**Conclusions and Relevance:**

This study evaluated the potential of administrative data in determining the optimal timing of PPS implementation. The quarterly PPS balanced precision and sustainability, especially when implemented during strategically selected periods across different seasons. Further studies are needed to validate the algorithm used in this study, especially in post–COVID-19 pandemic years and different settings.

## Key Points

**Question:**

How often should point prevalence surveys (PPSs) be conducted to accurately track antibiotic use among hospitalized children?

**Findings:**

In this prognostic study of 479 966 antibiotic prescriptions in pediatric inpatient and outpatient settings in Italy, quarterly data collection at specific times throughout the year gave a more accurate picture of antibiotic use than the current yearly surveys. Optimal data collection times from outpatient settings also worked well for inpatient settings, suggesting a way to streamline data collection.

**Meaning:**

This study highlights the potential of administrative data to help identify the optimal timing and periods for conducting PPSs to assess inpatient antibiotic use accurately.

## Teaser

This prognostic study explores the optimal frequency and timing of point prevalence surveys to collect data on antibiotic use among the pediatric population in inpatient and outpatient settings.

## Introduction

Antibiotics are among the most commonly prescribed agents globally, particularly in childhood.^1,2^ A clear and representative overview of antibiotic use is essential for guiding targeted and effective health policies.^3^ Health care and administrative databases are vital for systematically collecting local, regional, and national antibiotic prescription and consumption data. These tools allow investigators to assess the effectiveness of antimicrobial stewardship programs and identify areas for improvement.^4,5^ However, electronic data collection is not always accessible, particularly in smaller facilities, and manual data collection may be required.

Point prevalence surveys (PPSs) provide a snapshot of antibiotic use within a population at a single time point, showing prevalence but lacking data on temporal trends.^6^ However, the optimal frequency of PPS implementation remains undetermined. The World Health Organization^7^ and the Global PPS Study^8^ have used a yearly PPS to estimate antibiotic use among inpatients. To our knowledge, the accuracy of the yearly PPS approach, the most opportune timing for data collection, and its generalizability to smaller health care settings (eg, individual hospital departments) have yet to be rigorously investigated.

In this study, we aimed to develop an algorithm for identifying optimal time frames for collecting antibiotic use data within the calendar year. The algorithm specifically focused on maximizing data informativeness while minimizing resource expenditure by evaluating changes associated with varying data collection frequencies within these strategic periods. Furthermore, we investigated the applicability of optimal PPS time frames obtained from the outpatient setting to the inpatient setting within the same geographic region.

## Methods

This prognostic study was conducted in accordance with the Declaration of Helsinki^9^ and complied with European Network of Centres for Pharmacoepidemiology and Pharmacovigilance methodological standards in pharmacoepidemiology.^10^ Regarding outpatient data, the Internal Scientific Committee of Società Servizi Telematici Srl, the legal owner of Pedianet, granted ethical approval and access to the Pedianet database. Written informed consent was provided by participants’ legal guardians or next of kin. Regarding inpatient data, the Padua University Hospital Institutional Review Board approved this study. Informed consent was waived due to the study’s retrospective nature. The study followed the Standards for Reporting of Diagnostic Accuracy Studies (STARD) reporting guideline.

### Study Design, Study Population, and Data Source

This study applied a cross-sectional validation approach based on data collected from inpatient and outpatient databases for the Veneto region of Italy. The study cohorts were established based on dates of all antibiotic prescriptions for patients aged younger than 15 years admitted to the Padua University Hospital Pediatric Acute Care Unit (PACU) (inpatient setting) and patients who received care from family pediatricians in the Veneto-Pedianet network (outpatient setting).

Padua University Hospital provides primary and secondary care to 350 000 individuals (of which, 45 000 are aged <15 years) in a metropolitan area and tertiary care to the regional and extraregional population. There are approximately 25 000 visits per year to the Pediatric Emergency Department and 1000 admissions per year to the PACU.

Clinical, demographic, diagnostic, and daily prescription data for the inpatient setting were collected manually from electronic medical records for October 1, 2014, to December 31, 2022, using a password-protected RedCap data collection form. All data were stored on the secured server at Padua University. To guarantee anonymity and privacy, a unique study-specific number was assigned to each patient.

Outpatient data were retrieved from the Pedianet database for January 1, 2010, to December 31, 2022. Pedianet is an independent network of more than 200 family pediatricians who use an established pediatric primary care database based on JuniorBit software (Società di Servizi per la Pediatria) in their clinical practice.^11^ The network covers approximately 15% of the Veneto pediatric population and has previously been described in detail.^12^ Data generated by Pedianet family pediatricians are anonymized, in compliance with Italian regulations, stored in a protected cloud under a unique numeric identifier, and regularly checked for validation and quality control. Pedianet records several types of patient-level information, including demographic data, health status, and drug prescriptions. The representativeness of Pedianet data was evaluated elsewhere.^13^

### Outcomes

In this study, antibiotics were classified according to the 2023 World Health Organization (WHO) Access, Watch, and Reserve (AWARE) classification groups.^14,15^ Access antibiotics (eg, amoxicillin, amoxicillin-clavulanate) have a narrow spectrum, fewer side effects, and a lower risk of promoting resistance, and they are recommended as first-line treatment for common infections. Watch antibiotics (eg, second- or third-generation cephalosporins or macrolides), with a higher risk of promoting resistance, are typically used in more serious cases and require careful monitoring. Reserve antibiotics are last-resort options, reserved for severe infections caused by multidrug-resistant pathogens.^15^ Given that reserve antibiotics are seldom used in pediatrics (particularly in the outpatient setting), we decided to focus our analysis on the access and watch groups. Therefore, because their prevalence trends mirror each other, we exclusively analyzed access antibiotics.

For analysis, we used the epidemiologic year (eg, from September 23 of one year to September 22 of the following year). Years 2020 to 2022 were not considered in developing the proposed algorithm because the COVID-19 pandemic affected the antibiotic prescription pattern.^16^ However, we considered all available years for validation.

### Method to Identify the Highest Concordance Period to Collect Data

In this study, yearly PPS indicates a single data collection conducted annually; quarterly PPS denotes 4 distinct data collections, each carried out once per season (autumn, winter, spring, and summer) within a year. This time frame was chosen based on the results of our exploratory analysis (eMethods in Supplement 1), because it balanced the effort involved in gathering data with acceptable prognostic performance of annual antibiotic use, capturing seasonal trends, and reducing the data collection load.

The process was applied to all epidemiologic years (eg, between September 23 of one year and September 22 of the following year + n, where n is the number of available years). For yearly and quarterly PPSs, the following steps were performed independently for the inpatient and outpatient datasets each year.

The optimal day (±7 days) for a yearly PPS (eFigure 1 in Supplement 1) was identified as follows. First, we identified the day and its position in each epidemiologic year (eg, where September 23 of the first year covered the first position), which maximized concordance (in percentage points) between the daily access antibiotic prescribing rate (ie, Concordance = Number of Access Prescriptions/Total Number of Antibiotic Prescriptions) and the specific annual access antibiotic prescribing rate. Second, we weighted each position identified in step 1 by the number of prescriptions recorded each year on the overall number of prescriptions for the whole period to identify the optimal day and its position. Third, we included a lag (±7 days) from the day selected in step 2. Fourth, we calculated the daily access antibiotic prescribing rate in the 15-day period resulting from step 3. Fifth, we calculated the percentage point change (Δ) between each of the 15 daily access antibiotic prescribing rates resulting from step 4 and the annual access antibiotic prescribing rate. Finally, we calculated the mean change among the 15 Δ values from step 5 to build the 95% CI of the Δ distribution.

The 4 optimal days (±7 days) for the quarterly PPS were identified as follows (eFigure 2 in Supplement 1). First, we partitioned each epidemiologic year based on seasonality as follows: autumn (September 23 to December 20), winter (December 21 to March 19), spring (March 20 to June 20), and summer (June 21 to September 22). Second, we identified the day and the position in each season, which maximized concordance (in percentage points) between the daily access antibiotic prescribing rate and the seasonal access antibiotic prescribing rate. Third, we weighted each position identified in step 2 by the number of prescriptions recorded each season on the overall number of prescriptions for the whole period in each season to identify the optimal days and their position. Fourth, we included a lag (±7 days) from each of the 4 calendar days selected from point 3. Fifth, we calculated the daily access antibiotic prescribing rate in the 15-day period for each season resulting from point 4. Sixth, we calculated the percentage point change (Δ) between each of the 15^4^ daily access antibiotic prescribing rate combinations calculated in point 5 and the annual access antibiotic prescribing rate for each year. Finally, we calculated the mean change among the 15^4^ Δ values calculated from point 6 each year to build the 95% CI of the Δ distribution.

### Statistical Analysis

The distributions of sex (male or female), comorbidities (prematurity, cardiovascular diseases, once-hematologic conditions, chronic neurologic conditions, chronic respiratory conditions, chronic gastrointestinal conditions, or genetic diseases), and age at prescription date between the inpatient and outpatient settings were analyzed with frequencies (percentages) and medians (IQRs). A χ^2^ test and a Wilcoxon rank sum test were used to assess differences between the 2 populations. *P* < .05 (2-sided) was considered significant.

To evaluate the accuracy of the index test (ie, quarterly PPS) and the standard (ie, yearly PPS) in representing the annual access antibiotic prescribing rate, the mean Δ value (expressed as the percentage point difference between the mean yearly or quarterly access antibiotic prescribing rates of each year with annual access antibiotic prescribing rate of the same year with 95% CI) was calculated for each epidemiologic year. We used the concordance correlation coefficient (with the Kendall rank correlation test) to measure agreement between quarterly and yearly PPSs in representing annual antibiotic use in both inpatient and outpatient settings, considering both correlation and consistency.

To evaluate whether an outpatient database could be informative of the periods in which to collect data in an inpatient setting, we performed yearly and quarterly PPSs within the periods identified through the outpatient dataset with the inpatient dataset. The mean Δ values obtained were compared with the corresponding end points calculated previously.

All statistical analyses were performed using SAS, version 9.4 (SAS Institute Inc). Data analysis was performed from October 2023 to January 2024.

## Results

### Cohort Description

A total of 106 309 children were included in the analysis. There were 3124 inpatients (1351 females [43.2%] and 1773 males [56.8%]), with a median age of 2.6 (IQR, 0.6-6.6) years. There were 103 185 outpatients (49 534 females [48.0%] and 53 651 males [52.0%]), with a median age of 3.2 (IQR, 1.3-6.3) years. Considering that a child could have had more than 1 prescription at the same time or different prescriptions for different infectious episodes, information about 479 966 antibiotic prescriptions was collected; of these, 474 867 were from outpatients and 5099 were from inpatients (eTables 1 and 2 in Supplement 1).

Children presented different sociodemographic and clinical characteristics between the 2 cohorts (eTable 1 in Supplement 1). Specifically, the inpatient children were younger than the outpatient children (median age, 2.6 [IQR, 0.6-6.6] years vs 3.2 [1.3-6.3] years; *P* < .001) and had a higher burden of clinical comorbidities (≥1 comorbidity: 1141 of 3124 [36.5%] vs 6618 of 103 185 [6.4%], respectively; *P* < .001).

Antibiotic prescribing practice varied from outpatient to inpatient settings, with a higher prevalence of access antibiotic prescriptions in the former. According to age at prescription, the younger the child, the higher the access antibiotic prescribing rates. For example, among children aged 0 to 2 years and 12 years or older, rates were 83 687 of 117 073 (71.5%) and 17 653 of 34 973 (50.5%) for the outpatient setting and 1358 of 2417 (56.2%) and 230 of 635 (36.2%) for the inpatient setting, respectively. The same trend was observed between healthy children and children with comorbidities. No sex differences were observed in the AWARE categories in either setting (eFigure 3 and eTable 2 in Supplement 1).

### Evaluation of the Highest Concordance Periods and Frequency of PPS Implementation

#### Inpatient Setting

**Figure 1.**
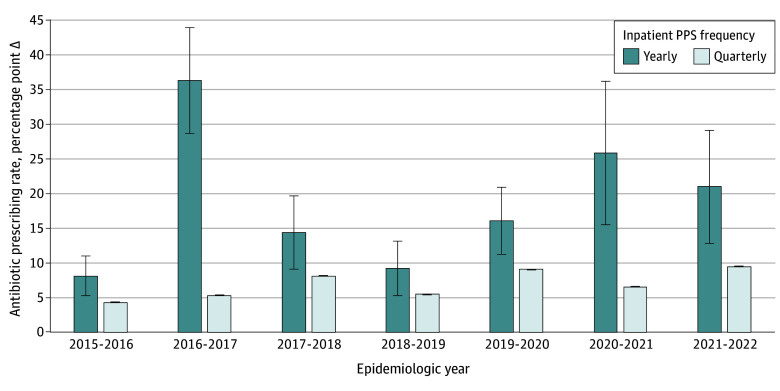
Yearly and Quarterly Point Prevalence Surveys (PPSs) in the Inpatient Setting Error bars represent 95% CIs.

According to annual antibiotic prescribing rates, use of access antibiotics in the inpatient setting increased from 37.5% in 2015 to 54.9% in 2022. For the yearly PPS, the algorithm selected the 15 days from October 20 to November 3 as the optimal period. The mean Δ ranged from 8.1 (95% CI, 5.3-11.0) to 36.4 (95% CI, 28.7-44.0) percentage points compared with the annual access antibiotic prescribing rate of the same year (Figure 1 and eTable 3 in Supplement 1).

For the quarterly PPS, the selected 15-day periods for each season were from (1) October 14 to October 28 in autumn, (2) December 30 to January 13 in winter, (3) April 6 to April 20 in spring, and (4) July 17 to July 31 in summer. Notably, the mean Δ difference in antibiotic prescribing rates compared with the yearly PPS was consistently lower, ranging from 4.3 (95% CI, 4.27-4.33) to 9.5 (95% CI, 9.41-9.54) percentage points relative to the yearly access antibiotic prescribing rate for the corresponding year. This finding translated to capturing 40.5% to 85.4% more of the overall annual access antibiotic prescribing rate by using the quarterly PPS method compared with the yearly PPS approach; that is, Year Point Prevalence = (Δ Yearly PPS − Δ Quarterly PPS)/Δ Yearly PPS (Figure 1 and eTable 3 in Supplement 1). Furthermore, we found significantly greater agreement of the annual inpatient access antibiotic prescribing rate for the quarterly PPS (*r* = 0.17, *P* < .001) compared with the yearly PPS (*r* = 0.04, *P* = .58) (Table).

**Table.** Correlation Between Yearly and Quarterly PPSs With Year Point Prevalence

PPS implementation by setting	Kendall correlation	*P* value
Inpatient		
Yearly	0.04	.58
Quarterly	0.17	<.001
Outpatient		
Yearly	0.05	.34
Quarterly	0.42	<.001

Abbreviation: PPS, point prevalence survey.

#### Outpatient Setting

According to overall annual access antibiotic prescribing rates, use of access antibiotics in the outpatient setting increased from 58.7% in 2010 to 65.1% in 2022. In contrast, the algorithm selected a 15-day period for the yearly PPS between June 14 and June 28 to collect data. The mean Δ difference in antibiotic prescribing rates ranged from approximately 13.2 (95% CI, 2.05-24.27) to 24.8 (95% CI, 12.55-37.08) percentage points compared with the true annual rate.

**Figure 2.**
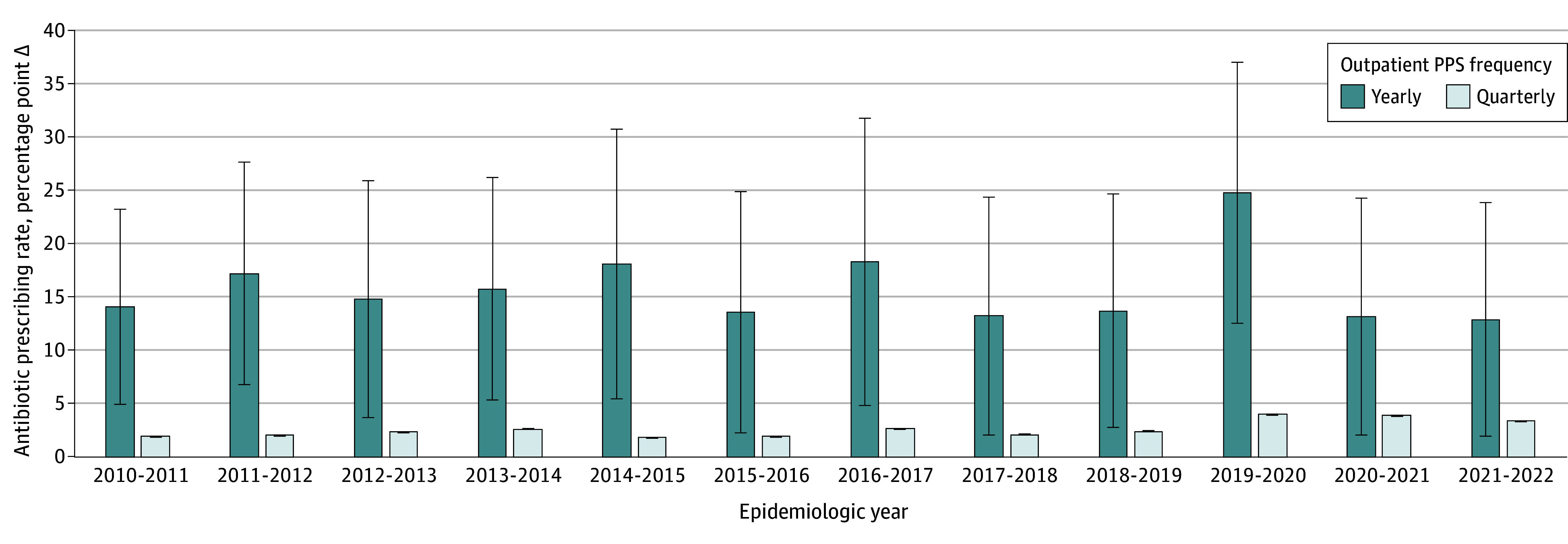
Yearly and Quarterly Point Prevalence Surveys (PPSs) in the Outpatient Setting Error bars represent 95% CIs.

The 4 time periods identified for the quarterly PPS were as follows: (1) from October 12 to October 26 in autumn, (2) from January 27 to February 10 in winter, (3) from May 9 to May 23 in spring, and (4) from July 24 to August 7 in summer. The mean Δ difference ranged from 1.9 (95% CI, 1.8-1.9) to 4.0 (95% CI, 3.9-4.0) percentage points to the annual access antibiotic prescribing rate of the same year (Figure 2 and eTable 4 in Supplement 1), while a positive agreement was not found with the yearly PPS (*r* = 0.05, *P* = .34).

### Application of the Highest Concordance Periods to Implement a PPS From the Outpatient to Inpatient Setting

The highest concordance period obtained from the outpatient dataset was applied to the inpatient setting, and the results were similar. The mean Δ between the quarterly PPS access antibiotic rate and the overall annual access antibiotic rate was consistently lower than that resulting from the yearly PPS, capturing 2.4% to 82.4% more of the overall annual access antibiotic prescribing rate by using the quarterly PPS method compared with the yearly PPS approach (eTable 5 in Supplement 1), with a Δ reduction of up to 89.8%. In our scenario, the generalizability of the optimal PPS periods identified from the outpatient setting to the inpatient setting was confirmed by the minimal difference in mean Δ values observed. In other words, applying the outpatient-derived periods to the inpatient data yielded results consistent with the aforementioned findings (eTable 6 in Supplement 1).

## Discussion

This study explored the optimal frequency of PPS implementation to collect data on antibiotic use among the pediatric population. Collecting antibiotic data is a crucial step in assessing the efficacy of a stewardship intervention.^17^ Although daily data collection is optimal, it is time-consuming and wastes human resources. Point prevalence surveys have emerged as a crucial tool for gathering information about antibiotic prescriptions with minimal effort.^6,18^ Determining the optimal timing for conducting PPSs in a year is essential to strike a balance between extensive data collection and an undue burden on health care systems. Infrequent surveys may overlook or overestimate dynamic changes, whereas too frequent surveys may lead to fatigue and compromise data quality.^19,20,21^

The 1-day PPS is the widespread method used to collect data on antibiotic prescriptions worldwide.^22,23^ In our study, the yearly PPS provided a less accurate approximation of annual access antibiotic prescribing rates compared with the quarterly PPS approach, although the data were collected in the highest concordance periods identified by the algorithm and not on random days. Our results support the hypothesis that the quarterly PPS may be an optimal tool for monitoring antibiotic prescriptions, combining practicality and precision, especially when conducted during preidentified specific periods in different seasons.

Determining the optimal periods for PPSs is challenging. Seasonal variations and epidemic fluctuations can differ across years and locations, making it difficult to identify optimal data collection timing. Our analysis revealed an unexpected result: June, a summer month usually associated with lower antibiotic use, provided estimates closest to the annual outpatient antibiotic access rates. This finding suggests that the typical winter peak in antibiotic prescribing may not accurately represent the true annual quality of prescriptions. Watch antibiotics, reserved for more serious infections and subject to stricter stewardship, may contribute to this discrepancy. Their increased winter prescriptions may distort the perceived annual quality, highlighting the need for more nuanced data collection strategies. This study demonstrates the value of using administrative data from the outpatient setting to strategically define PPS periods for the inpatient setting. This approach may be advantageous from several points of view. First, the efficiency of the system in analyzing outpatient data enables identification of optimal periods for describing antibiotic prescribing activity, potentially decreasing the need for extensive data collection efforts within the inpatient setting. Second, if outpatient and inpatient antibiotic prescribing patterns exhibit similar seasonal trends, outpatient-derived optimal periods may potentially have external validity in the inpatient setting; this eliminates the need for separate period identification in each environment, saving resources. Third, by leveraging outpatient data, the burden of additional data collection within the inpatient setting can be minimized. Although this study suggests the potential applicability of outpatient-derived PPS periods, some validation within the inpatient setting may be necessary. The specific characteristics of each health care facility and patient population may require further customization. This rephrasing emphasizes the strategic benefits of using outpatient data to define PPS periods for inpatient evaluation, and it highlights the potential for increased efficiency, applicability, and reduced burden while acknowledging the need for further validation and customization.

### Limitations

This study has some limitations. Antibiotic prescription patterns during the COVID-19 pandemic years may have been different compared with the previous and following years. In 2020 and early 2021, the incidence of respiratory tract infections decreased, which is plausible due to the restrictive measures implemented globally.^24^ However, respiratory tract infections resurged from the second half of 2021, marked by an early onset of viral epidemics.^25^ It is possible that the strategic period identified with our algorithm using prepandemic data may not be suitable for the postpandemic years. New specific periods may be necessary, and our algorithm should be retrained in the upcoming years to identify new strategic periods for data collection unless seasonal epidemics revert to their patterns before the COVID-19 pandemic.

Adding a lag time (±7 days) may have introduced bias in assessing the optimal days to collect antibiotic prescriptions. It is impossible to identify a specific day to collect data due to high variabilities in the number of visits per day, the number of prescriptions per day, and weekends and vacations. However, limiting data collection to a specific 15-day period is a step toward improving PPS planning and implementation. Finally, the study population was exclusively pediatric, limiting the generalizability of these findings to other age groups.

## Conclusions

The findings of this prognostic study suggest that PPSs, when conducted quarterly and strategically timed throughout the year, may provide a good balance between precision and sustainability for capturing antibiotic use patterns. Additionally, administrative data may be used to determine optimal PPS timing in the inpatient setting. The use of an algorithm applied to administrative data was effective in identifying strategic time periods for accurate data collection. This approach offers a potentially resource-efficient strategy for optimizing PPS timing in the inpatient setting. This study also highlighted the valuable interplay between data from different health care settings, ultimately contributing to more informed antibiotic stewardship efforts. Nevertheless, further studies are needed to validate the algorithm used here, especially in the post–COVID-19 pandemic years and different settings.

## Notes

Supplement 1. **eMethods.** Exploratory Analysis **eTable 1.** Distribution of Children at First Prescription According to Age (Years), Sex, and Comorbidities **eTable 2.** Prescriptions Distribution According to Age (Years), Sex, and Comorbidities **eTable 3.** Inpatient Delta Distribution With Inpatient Selected Date for Yearly and Quarterly Point Prevalence Surveys **eTable 4.** Outpatient Delta Distribution With Outpatient Selected Date for Yearly and Quarterly Point Prevalence Surveys **eTable 5.** Inpatient Delta Distribution With Outpatient Selected Date for Yearly and Quarterly Point Prevalence Surveys **eTable 6.** Comparison Between Inpatient and Outpatient Quarterly Point Prevalence Survey Date in the Inpatient Setting **eFigure 1.** Yearly Point Prevalence Survey Calculation Algorithm **eFigure 2.** Quarterly Point Prevalence Survey Calculation Algorithm **eFigure 3.** AWARE Prescription Distribution According to Outpatient and Inpatient Settings, Stratified by Comorbidities, Sex, and Age at Prescription

Supplement 2.
Data Sharing Statement
