# Supplementary material for: Validation of Administrative Data and Timing of Point Prevalence Surveys for Antibiotic Monitoring
Source: JAMA Netw Open. 2024 Sep 24;7(9):e2435127. doi: 10.1001/jamanetworkopen.2024.35127 (PMC11423167; doi:10.1001/jamanetworkopen.2024.35127)
Supplement: Supplement 1. — eMethods. Exploratory Analysis eTable 1. Distribution of Children at First Prescription According to Age (Years), Sex, and Comorbidities eTable 2. Prescriptions Distribution According to Age (Years), Sex, and Comorbidities eTable 3. Inpatient Delta Distribution With Inpatient Selected Date for Yearly and Quarterly Point Prevalence Surveys eTable 4. Outpatient Delta Distribution With Outpatient Selected Date for Yearly and Quarterly Point Prevalence Surveys eTable 5. Inpatient Delta Distribution With Outpatient Selected Date for Yearly and Quarterly Point Prevalence Surveys eTable 6. Comparison Between Inpatient and Outpatient Quarterly Point Prevalence Survey Date in the Inpatient Setting eFigure 1. Yearly Point Prevalence Survey Calculation Algorithm eFigure 2. Quarterly Point Prevalence Survey Calculation Algorithm eFigure 3. AWARE Prescription Distribution According to Outpatient and Inpatient Settings, Stratified by Comorbidities, Sex, and Age at Prescription [file jamanetwopen-e2435127-s001.pdf]

Supplementary Online Content

Boracchini R, Brigadoi G, Barbieri E, et al. Validation of administrative data and timing of point prevalence surveys for antibiotic monitoring. *JAMA Netw Open*. 2024;7(9):e2435127. doi:10.1001/jamanetworkopen.2024.35127

eMethods. Exploratory Analysis

eTable 1. Distribution of Children at First Prescription According to Age (Years), Sex, and Comorbidities

eTable 2. Prescriptions Distribution According to Age (Years), Sex, and Comorbidities

eTable 3. Inpatient Delta Distribution With Inpatient Selected Date for Yearly and Quarterly Point Prevalence Surveys

eTable 4. Outpatient Delta Distribution With Outpatient Selected Date for Yearly and Quarterly Point Prevalence Surveys

eTable 5. Inpatient Delta Distribution With Outpatient Selected Date for Yearly and Quarterly Point Prevalence Surveys

eTable 6. Comparison Between Inpatient and Outpatient Quarterly Point Prevalence Survey Date in the Inpatient Setting

eFigure 1. Yearly Point Prevalence Survey Calculation Algorithm

eFigure 2. Quarterly Point Prevalence Survey Calculation Algorithm

eFigure 3. AWARE Prescription Distribution According to Outpatient and Inpatient Settings, Stratified by Comorbidities, Sex, and Age at Prescription

This supplementary material has been provided by the authors to give readers additional information about their work.

© 2024 Boracchini R et al. *JAMA Network Open.*

eMethods. Exploratory Analysis

Prior to algorithm development, an exploratory pre-assessment analysis assessed the suitability of various periodicities for predicting antibiotic use starting from a pre-specified random day of the year. Absolute differences were calculated between annual and predicted antibiotic prescribing rates (yearly, quarterly, bimonthly, monthly, twice- and four times a month).

After 6 annual measurements, the absolute difference with the annual prescribing rate measure flattened out, showing nearly overlapping values. However, this would require a significant data collection effort. While yearly and quarterly models yielded different prediction performances, the quarterly model demonstrated superior performance in all five evaluation metrics. Consequently, we chose quarterly-PPS as the index test compared to the yearly-PPS (gold standard). Quarterly periodicity was selected for subsequent algorithm development, as it was hypothesized to better capture fluctuations in antibiotic use through the incorporation of more granular temporal data.

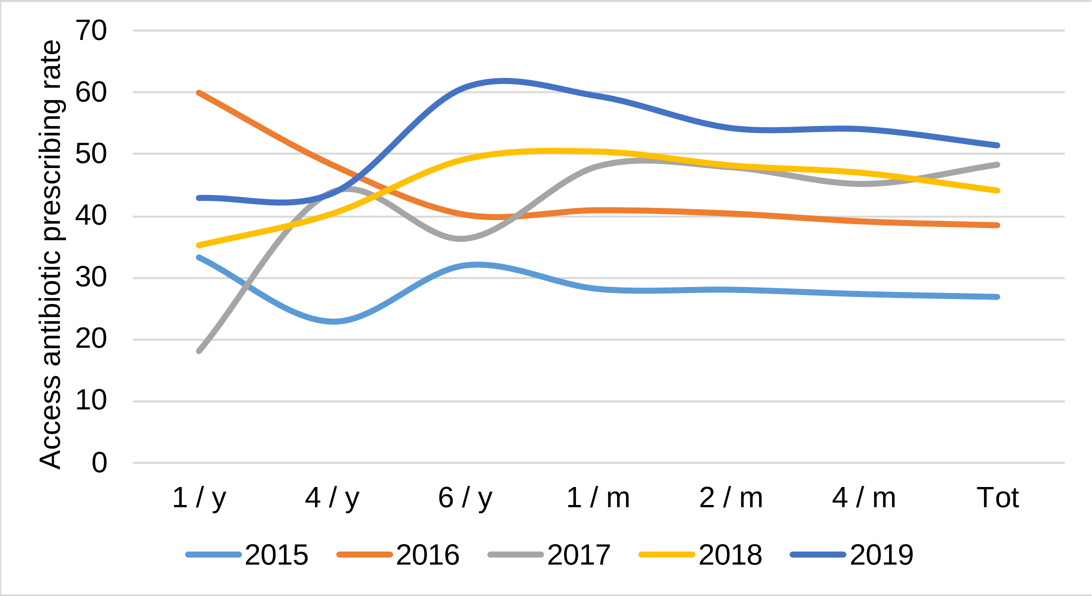

Various periodicities for predicting access antibiotic prescribing rate compared to the annual access prescribing rates

© 2024 Boracchini R et al. *JAMA Network Open.*

eTable 1. Distribution of Children at First Prescription According to Age (Years), Sex, and Comorbidities

	Inpatient (N = 3124)	Outpatient (N = 103 185)	P-value[a]
Age in years, median (p25 - p75)	2.56 (0.57 - 6.62)	3.15 (1.27 - 6.31)	<.0001
0 - 2	1547 (49.5)	43 696 (42.4)	<.0001
3 - 5	497 (15.9)	22 152 (21.5)	
6 - 11	729 (23.3)	30 565 (29.6)	
≥ 12	351 (11.2)	6772 (6.6)	
Sex			
Female	1351 (43.3)	49 534 (48.0)	<.0001
Male	1773 (56.8)	53 651 (52.0)	
Comorbidities[b]			
0	1983 (63.5)	96 567 (93.6)	<.0001
At least 1	1141 (36.5)	6618 (6.4)	

a, Chi-Squared test and a Wilcoxon Rank Sum test as appropriate.
b, Health impairment warranting exemption

© 2024 Boracchini R et al. *JAMA Network Open.*

eTable 2. Prescriptions Distribution According to Age (Years), Sex, and Comorbidities

	Overall			Access			Watch		
	Inpatient (N=5099)	Outpatient (N = 474 867)	P-value[a]	Inpatient (N = 2460)	Outpatient (N = 303 386)	P-value[a]	Inpatient (N = 2639)	Outpatient (N = 171 481)	P-value[a]
Age in years, median (p25 - p75)	2.84 (0.57 - 7.07)	4.36 (2.53 - 7.19)	<.0001	1.88 (0.22 - 5.78)	4.10 (2.28 - 6.62)	<.0001	3.71 (1.06 - 8.58)	4.87 (3.01 - 8.20)	<.0001
0 - 2	2417 (47.4)	117 073 (24.7)	<.0001	1358 (55.2)	83 687 (27.6)	<.0001	1059 (40.1)	33 386 (19.5)	<.0001
3 - 5	795 (15.6)	128 542 (27.1)		362 (14.7)	83 876 (27.7)		433 (16.4)	44 666 (26.1)	
6 - 11	1252 (24.6)	194 279 (40.9)		5410 (20.7)	118 170 (39.0)		742 (28.1)	76 109 (44.4)	
≥ 12	635 (12.5)	34 973 (7.4)		230 (9.4)	17 653 (5.8)		405 (15.4)	17 320 (10.1)	
Sex									
Female	2191 (43.0)	224 260 (47.2)	<.0001	1052 (42.8)	144 198 (47.5)	<.0001	1139 (43.2)	80 062 (46.7)	0.0003
Male	2908 (57.0)	250 607 (52.8)		1408 (57.2)	159 188 (52.5)		1500 (56.8)	91 419 (53.3)	
Comorbidities									
0	3050 (59.8)	432 289 (91.0)	<.0001	1679 (68.3)	278 027 (91.6)	<.0001	1371 (52.0)	154 262 (90.0)	<.0001
At least 1	2049 (40.2)	42 578 (9.0)		781 (31.8)	25 359 (8.4)		1268 (48.1)	17 219 (10.0)	

a, Chi-Squared test and a Wilcoxon Rank Sum test as appropriate.

© 2024 Boracchini R et al. *JAMA Network Open.*

eTable 3. Inpatient Delta Distribution With Inpatient Selected Date for Yearly and Quarterly Point Prevalence Surveys

	YPP (%)	Yearly-PPS	Quarterly-PPS	Delta reduction (%)
2015/2016	37.5	8.12 (5.27 - 10.97)	4.3 (4.27 - 4.33)	3.82 (47.0)
2016/2017	45.6	36.35 (28.71 - 43.99)	5.3 (5.27 - 5.34)	31.05 (85.4)
2017/2018	47.2	14.4 (9.15 - 19.64)	8.09 (8.05 - 8.14)	6.31 (43.8)
2018/2019	48.7	9.2 (5.28 - 13.13)	5.47 (5.43 - 5.5)	3.73 (40.5)
2019/2020	45.2	16.13 (11.29 - 20.98)	9.09 (9.05 - 9.14)	7.04 (43.7)
2020/2021	52.1	25.88 (15.49 - 36.26)	6.53 (6.49 - 6.58)	19.35 (74.8)
2021/2022	54.9	21.01 (12.8 - 29.21)	9.47 (9.41 - 9.54)	11.54 (54.9)

Abbreviations: EY, Epidemiological Year; YPP, Year Point Prevalence; PPS, Point Prevalence Survey

eTable 4. Outpatient Delta Distribution With Outpatient Selected Date for Yearly and Quarterly Point Prevalence Surveys

	YPP (%)	Yearly-PPS	Quarterly-PPS	Delta reduction (%)
2010/2011	58.7	14.11 (4.9 - 23.31)	1.92 (1.9 - 1.93)	12.19 (86.4)
2011/2012	63.0	17.24 (6.78 - 27.69)	2.01 (1.99 - 2.03)	15.23 (88.3)
2012/2013	64.7	14.81 (3.65 - 25.98)	2.31 (2.3 - 2.33)	12.5 (84.4)
2013/2014	61.7	15.78 (5.29 - 26.27)	2.58 (2.55 - 2.6)	13.2 (83.7)
2014/2015	63.6	18.11 (5.39 - 30.83)	1.85 (1.83 - 1.87)	16.26 (89.8)
2015/2016	67.3	13.6 (2.23 - 24.98)	1.95 (1.93 - 1.97)	11.65 (85.7)
2016/2017	67.5	18.37 (4.86 - 31.88)	2.62 (2.6 - 2.63)	15.75 (85.7)
2017/2018	67.2	13.24 (2.07 - 24.41)	2.08 (2.06 - 2.1)	11.16 (84.3)
2018/2019	65.8	13.74 (2.75 - 24.73)	2.39 (2.37 - 2.41)	11.35 (82.6)
2019/2020	65.4	24.81 (12.55 - 37.08)	3.97 (3.94 - 4.01)	20.84 (84.0)
2020/2021	65.9	13.16 (2.05 - 24.27)	3.85 (3.82 - 3.88)	9.31 (70.7)
2021/2022	65.1	12.89 (1.89 - 23.89)	3.33 (3.3 - 3.35)	9.56 (74.2)

Abbreviations: EY, Epidemiological Year; YPP, Year Point Prevalence; PPS, Point Prevalence Survey

© 2024 Boracchini R et al. *JAMA Network Open.*

eTable 5. Inpatient Delta Distribution With Outpatient Selected Date for Yearly and Quarterly Point Prevalence Surveys

EY	Mean delta (95% CI)		Delta reduction (%)
	Yearly-PPS	Quarterly-PPS	
2015/2016	10.32 (6.67 - 13.97)	4.04 (4.02 - 4.07)	6.28 (60.9)
2016/2017	15.35 (10.31 - 20.38)	6.17 (6.13 - 6.21)	9.18 (59.8)
2017/2018	14.2 (10.08 - 18.33)	3.88 (3.85 - 3.9)	10.32 (72.7)
2018/2019	26.63 (15.68 - 37.58)	4.7 (4.67 - 4.73)	21.93 (82.4)
2019/2020	8.42 (5.36 - 11.49)	8.08 (8.03 - 8.12)	0.34 (4.0)
2020/2021	18.04 (10.76 - 25.33)	9.3 (9.24 - 9.36)	8.74 (48.5)
2021/2022	8.4 (5.82 - 10.98)	8.2 (8.14 - 8.25)	0.2 (2.4)

Abbreviations: EY, Epidemiological Year; YPP, Year Point Prevalence; PPS, Point Prevalence Survey

eTable 6. Comparison Between Inpatient and Outpatient Quarterly Point Prevalence Survey Date in the Inpatient Setting

EY	Inpatient best dates Quarterly-PPS Mean delta (95% CI)	Outpatient best dates Quarterly-PPS Mean delta (95% CI)
2015/2016	4.3 (4.27 - 4.33)	4.04 (4.02 - 4.07)
2016/2017	5.3 (5.27 - 5.34)	6.17 (6.13 - 6.21)
2017/2018	8.09 (8.05 - 8.14)	3.88 (3.85 - 3.9)
2018/2019	5.47 (5.43 - 5.5)	4.7 (4.67 - 4.73)
2019/2020	9.09 (9.05 - 9.14)	8.08 (8.03 - 8.12)
2020/2021	6.53 (6.49 - 6.58)	9.3 (9.24 - 9.36)
2021/2022	9.47 (9.41 - 9.54)	8.2 (8.14 - 8.25)

Abbreviations: EY, Epidemiological Year; YPP, Year Point Prevalence; PPS, Point Prevalence Survey

© 2024 Boracchini R et al. *JAMA Network Open.*

eFigure 1. Yearly Point Prevalence Survey Calculation Algorithm

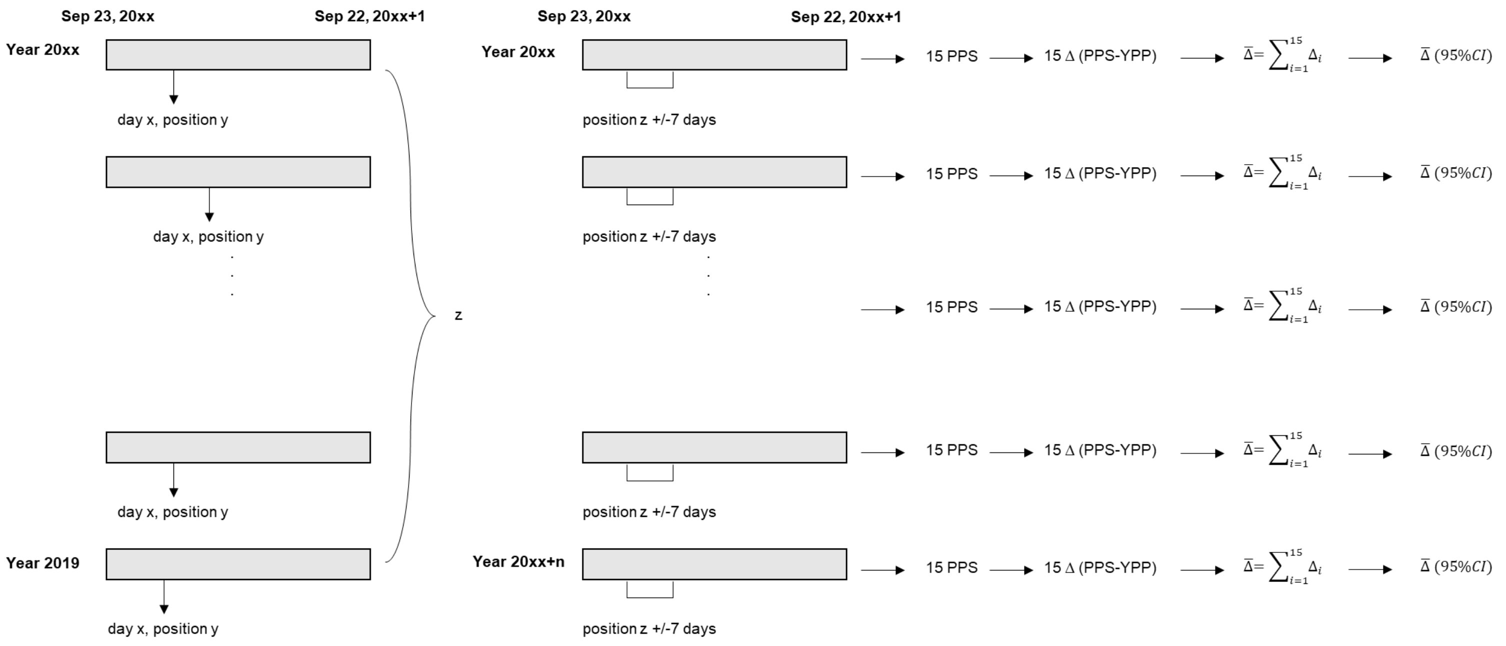

Abbreviations: PPS, Point Prevalence Survey; YPP, Year Point Prevalence; z, best position day weighted by the number of prescriptions recorded each year on the overall number of prescriptions forthe whole period (x)

© 2024 Boracchini R et al. *JAMA Network Open.*

eFigure 2. Quarterly Point Prevalence Survey Calculation Algorithm

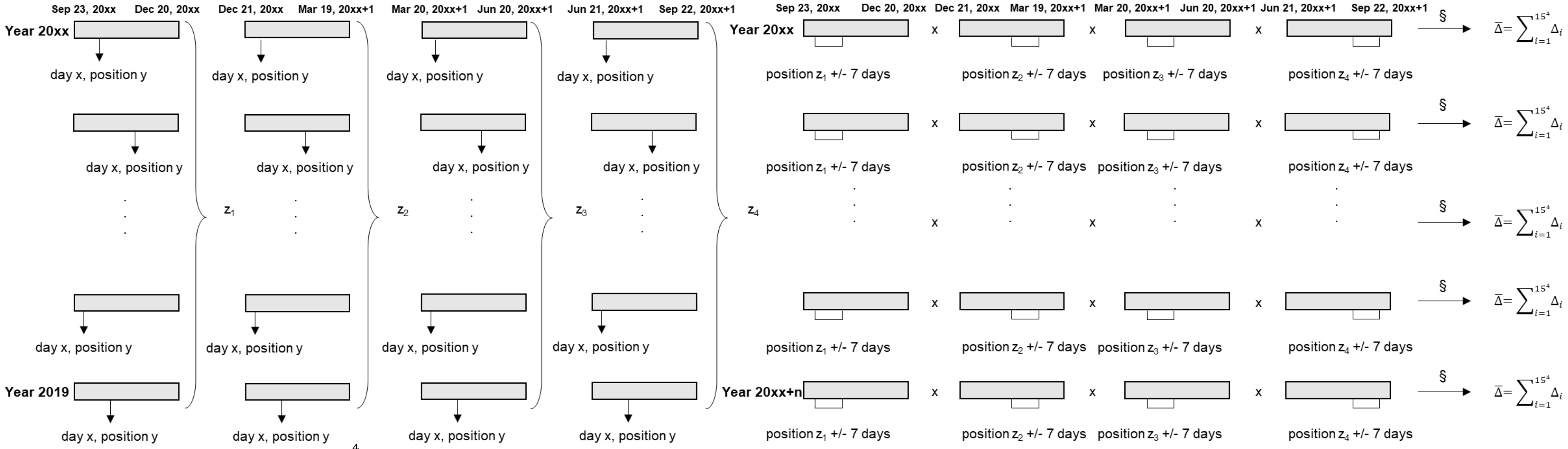

§, 15^4 $PPS \rightarrow 50\ 625\ \Delta(PPS - YPP) \rightarrow \bar{\Delta} = \sum_{i=1}^{15^4} \Delta_i \rightarrow \bar{\Delta}(95\%\ CI)$. Abbreviations: PPS, Point Prevalence Survey; YPP, Year Point Prevalence; z_i, best position day weighted by the number of prescriptions recorded each season on the overall number of prescriptions for the whole year.

© 2024 Boracchini R et al. *JAMA Network Open.*

eFigure 3. AWARE Prescription Distribution According to Outpatient and Inpatient Settings, Stratified by Comorbidities, Sex, and Age at Prescription

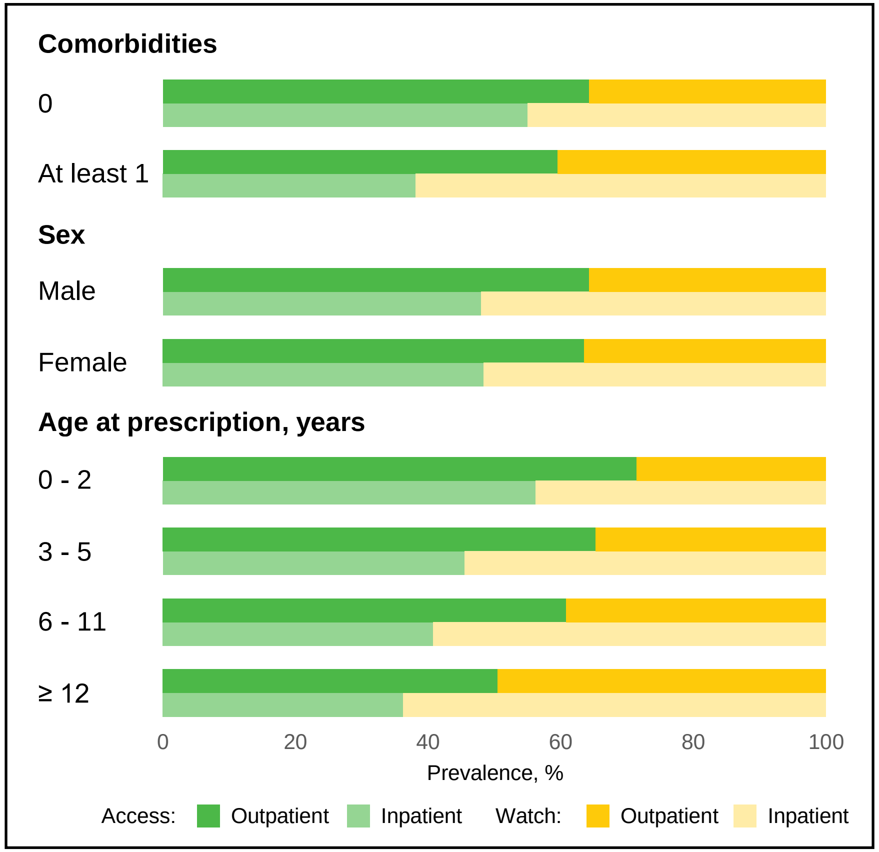

Abbreviations: AWARE, Access, Watch and Reserve

© 2024 Boracchini R et al. *JAMA Network Open.*
