# Supplementary material for: Validation of Administrative Data and Timing of Point Prevalence Surveys for Antibiotic Monitoring
Source: JAMA Netw Open. 2024 Sep 24;7(9):e2435127. doi: 10.1001/jamanetworkopen.2024.35127 (PMC11423167; doi:10.1001/jamanetworkopen.2024.35127)
Supplement: Supplement 2. — Data Sharing Statement [file jamanetwopen-e2435127-s002.pdf]

Data Sharing Statement

Boracchini. Validation of Administrative Data and Timing of Point Prevalence Surveys for Antibiotic Monitoring. *JAMA Netw Open*. Published September 24, 2024. doi:10.1001/jamanetworkopen.2024.35127

Data
Data available: No

Additional Information
Explanation for why data not available: The data used in this study cannot be made publicly available due to Italian data protection laws. The anonymized datasets generated during the current study can be provided on request, from the corresponding author, after written approval by the Internal Scientific Committee
